# Iron and leukemia: new insights for future treatments

**DOI:** 10.1186/s13046-019-1397-3

**Published:** 2019-09-13

**Authors:** Fang Wang, Huanhuan Lv, Bin Zhao, Liangfu Zhou, Shenghang Wang, Jie Luo, Junyu Liu, Peng Shang

**Affiliations:** 1 School of Life Science, Northwestern Polytechnical University, Xi’an, 710072 China; 2 Research & Development Institute of Northwestern Polytechnical University in Shenzhen, Shenzhen, 518057 China; 3 Key Laboratory for Space Bioscience and Biotechnology, Institute of Special Environment Biophysics, Northwestern Polytechnical University, Xi’an, 710072 China

**Keywords:** Leukemia, Iron, Reactive oxygen species, Ferroptosis, Iron-based nanoparticles

## Abstract

Iron, an indispensable element for life, is involved in all kinds of important physiological activities. Iron promotes cell growth and proliferation, but it also causes oxidative stress damage. The body has a strict regulation mechanism of iron metabolism due to its potential toxicity. As a cancer of the bone marrow and blood cells, leukemia threatens human health seriously. Current studies suggest that dysregulation of iron metabolism and subsequent accumulation of excess iron are closely associated with the occurrence and progress of leukemia. Specifically, excess iron promotes the development of leukemia due to the pro-oxidative nature of iron and its damaging effects on DNA. On the other hand, leukemia cells acquire large amounts of iron to maintain rapid growth and proliferation. Therefore, targeting iron metabolism may provide new insights for approaches to the treatment of leukemia. This review summarizes physiologic iron metabolism, alternations of iron metabolism in leukemia and therapeutic opportunities of targeting the altered iron metabolism in leukemia, with a focus on acute leukemia.

## Background

Iron is an indispensable nutrient. The maintenance of normal cell metabolism depends on iron. Iron enables the function of vital iron-containing enzymes that are involved in ATP production, DNA synthesis, oxygen transport and many other physiological activities. The ability of iron to gain and lose electrons enables it to participate in free radical generating reactions [1]. Among them is the Fenton reaction, in which ferrous iron (Fe^2+^) donates an electron to hydrogen peroxide to yield hydroxyl radical, a kind of highly invasive reactive oxygen species (ROS) [2]. ROS have effects on multiple cellular signaling pathways that are crucial for cell survival, proliferation and differentiation [3]. However, the aberrant accumulation of iron and subsequent excess ROS cause oxidative stress, which incurs damage to DNA, proteins, lipids or other biomolecules and even results in cell death [3]. Extensive researches have revealed links between dysregulation of iron metabolism and a number of diseases, including atherosclerosis, neurodegenerative diseases and cancer [4–6]. The oxidative effects of iron contribute to the oncogenesis and iron is essential for the development of cancer [7].

Leukemia is a group of heterogeneous hematopoietic stem cell (HSC) malignancies. It is characterized by aberrant accumulation of undifferentiated blasts capable of unrestrained proliferation in the bone marrow, which interferes with the production of normal blood cells. Leukemia is classified into four main subgroups, including acute myeloid leukemia (AML), acute lymphoblastic leukemia (ALL), chronic myeloid leukemia (CML) and chronic lymphoblastic leukemia (CLL). Leukemia, especially acute leukemia (AL), is one of the most common lethal cancers [8]. There is a general consensus that the occurrence of leukemia is a multistep process involving multiple genetic alterations, including transferrin receptor 1 gene, hemochromatosis (*HFE*) gene and some other genes involved in iron metabolism [9, 10]. Leukemia cells show increased iron uptake and decreased iron efflux, leading to elevated cellular iron levels. The systematic iron pool in patients with leukemia is also increased, which is aggravated by multiple red-blood-cell transfusions. Multiple experimental and epidemiological studies have demonstrated the relationship between dysregulation of iron metabolism with the occurrence and progress of leukemia [9–11].

Currently, the main approaches for clinical treatment of leukemia are chemotherapy and bone marrow transplantation. As leukemia cells are prevalent in the whole body and surrounded by normal blood cells, traditional chemotherapy drugs can also cause damage to healthy cells while killing leukemia cells. Although great progress has been made in recent years, the outcomes of patients with AL remain unsatisfactory and new therapeutic strategies are imperative to improve the outcomes of patients [12, 13]. The application of differentiating agents combined with chemotherapy has dramatically improved the therapeutic effect of patients with acute promyelocytic leukemia (APL). Accumulating evidence shows that targeting iron homeostasis can induce differentiation and apoptosis in leukemia cells [14–16]. Leukemia cells are dramatically more susceptible to iron depletion than normal cells due to their high requirement for iron to maintain their rapid proliferation. It has been evaluated that treatment targeting iron metabolism induces differentiation of leukemia cells without harm to normal cells [14]. Therefore, targeting iron metabolic pathways may be an optimal treatment which can selectively eradicate leukemia cells via multiple mechanisms. Here, we review physiologic iron metabolism, alternations of iron metabolism in leukemia, and therapeutic opportunities of targeting the altered iron metabolism in leukemia, with a focus on AL.

### Physiologic iron metabolism

Iron homeostasis is a complex and highly regulated process, which involves acquisition, utilization, storage and efflux of iron. Non-heme iron in the diet are mostly presented in the form of ferric iron (Fe^3+^) [17]. The absorption of non-heme iron in the diet involves reduction of Fe^3+^ to Fe^2+^ in the intestinal lumen by ferric reductases, such as duodenal cytochrome b reductase (Dcytb), and subsequent transport of Fe^2+^ into enterocytes by divalent metal transporter 1 (DMT1) [18]. Dietary heme iron can be directly taken up by enterocytes by a yet unknown mechanism [17]. The iron absorbed through enterocytes is either exported across the basolateral membrane into the circulation by ferroportin 1 (FPN1), the only known mammalian iron exporter, or stored in ferritin [19]. On the basolateral membrane, Fe^2+^ is oxidized by ferroxidase hephaestin (HEPH) in order to be associated with transferrin (Tf) in the plasma [20]. Iron is circulated throughout the body in a redox-inert state and is primarily utilized for erythropoiesis [21]. Senescent red blood cells are cleared by macrophages and the iron is released into the systemic iron pool [21]. The balance of whole-body iron is maintained by strictly regulating the absorption of dietary iron in the duodenum, which is mainly achieved by the ferroportin–hepcidin regulatory axis [22]. When whole-body iron levels are high, hepcidin is induced in hepatocytes and secreted into the circulation. Hepcidin binds to FPN1 on enterocytes and macrophages to block the delivery of iron into the circulation [23].

Tf-bound iron in the plasma can be taken up by cells mainly through transferrin receptor 1 (TfR1, 24]. Diferric Tf binds to TfR1 on the plasma membrane and the Tf/TfR1 complex is subsequently taken into the cell by receptor-mediated endocytosis [24]. In the endosome, iron is released from the complex [25], reduced by six-transmembrane epithelial antigen of the prostate (STEAP) proteins to Fe^2+^ and transported into the cytoplasm by DMT1 [26]. Meanwhile, the apo-transferrin (apo-Tf)/TfR1 complex is recycled to the cell surface where apo-Tf is released to the plasma. Certain types of cells can absorb iron in other forms such as non-transferrin bound iron (NTBI), ferritin, heme and hemoglobin [20]. Imported iron enters the cytosolic labile iron pool (LIP), a pool of chelatable and redox-active iron [27]. Iron in the pool is delivered to different parts of the cell for a variety of metabolic needs or stored in ferritin [28]. Excess cellular iron can be exported out of the cell by FPN1 and subsequently oxidized by the ceruloplasmin (Cp) and binded to serum Tf [29]. The cellular iron homeostasis is achieved mainly by the iron responsive elements (IREs)/ iron regulatory proteins (IRPs) system [30]. IRPs regulate the expression of genes involved in iron metabolism by binding to IREs. When cellular iron concentrations are low, the IRPs bind to the IREs, resulting in increased synthesis of TfR1 and decreased synthesis of ferritin and FPN1. This effect allows the cells to absorb iron to the utmost.

### Alternations of iron metabolism in leukemia

**Fig. 1:**
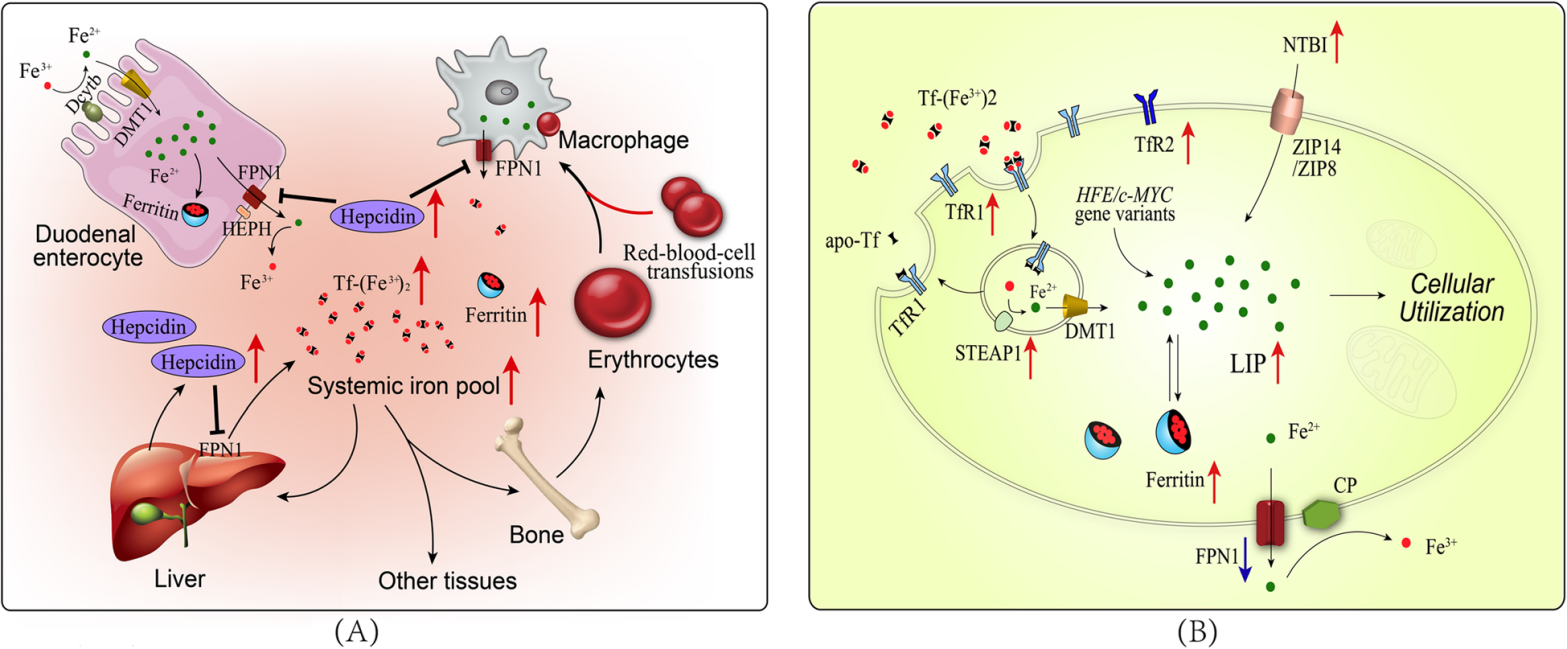
Alternations of iron metabolism in leukemia at systemic and cellular levels. **a** The systematic iron pool and serum ferritin levels are increased which is aggravated by multiple red-blood-cell transfusions. Hepcidin is induced to block the delivery of iron into the circulation from enterocytes, macrophages and some other cells. **b** Leukemia cells show increased iron uptake and decreased iron efflux, leading to elevated cellular iron levels. Proteins related to iron uptake such as TfR1, TfR2 and STEAP1 are overexpressed and absorption of NTBI is increased. However, the expression of iron export protein FPN1 is decreased. *HFE* or *c-MYC* gene variants are also associated with elevated intracellular iron levels in leukemia cells

Iron metabolism in leukemia is altered, including not only changes in cellular iron uptake, storage and efflux, but also dysregulation of the ferroportin–hepcidin regulatory axis (Fig. 1). Furthermore, multiple red-blood-cell transfusions throughout chemotherapy treatment aggravate systematic iron overload in patients with leukemia. While iron and its catalytic production of ROS are critical to maintain hematopoietic homeostasis, accumulation of iron and subsequent increased oxidative stress are detrimental to normal hematopoiesis. ROS have been implicated as the signal messengers in normal hematopoiesis and participate in controlling the biological activity of HSCs [31]. However, redox dysregulation caused by ROS promotes malignant transformation of HSCs by increasing DNA double strand breaks and repair errors [32, 33]. Besides, iron is essential for the progression of leukemia because maintaining the rapid growth rate of leukemia cells requires the iron-dependent enzyme ribonucleotide reductase for DNA synthesis [7, 34, 35]. Furthermore, iron overload allows leukemia cells immune evasion by triggering apoptosis of adjacent NK cells, CD4^+^ T cells and CD8^+^ T cells, but increasing percentage of regulatory T cells [36, 37].

### Alternations of iron metabolism in leukemia at systemic levels

It has been reported that patients with AML at diagnosis had higher levels of serum ferritin, the routine marker for excess iron [38]. Ferritin promotes the growth of leukemia cells while inhibiting the colony formation of normal progenitor cells, which is identified as leukemia-associated inhibitory activity [39]. Clinical analysis suggests that hyperferritinemia at diagnosis is significantly associated with chemotherapy drug resistance, a higher incidence of relapse as well as poorer overall survival [38, 40]. Furthermore, an elevated pretransplantation serum ferritin level is an adverse prognostic factor for overall survival and nonrelapse mortality for patients with hematologic malignancies undergoing allogeneic hematopoietic stem cell transplantation (allo-HSCT) [41, 42].

Due to the increased systematic iron pool, the ferroportin–hepcidin regulatory axis is also dysregulated. The serum hepcidin levels of AL patients are significantly elevated at the initial of diagnosis and decreased after remission, but still higher than that of the healthy controls [43, 44]. High level of serum hepcidin leads to iron accumulation in leukemia cells which may contribute to leukemogenesis by activating Wnt and nuclear factor kappa-B (NF-κB) signaling pathways [45–48].

Meanwhile, the transportation of iron into the circulation from enterocytes and macrophages is blocked, thereby leading to erythropoiesis suppression and iron accumulation in tissues. In addition, patients with AL usually receive multiple red-blood-cell transfusions for hematologic support, which aggravates systematic iron overload. Transfusional iron accumulates in macrophages initially as the senescent red blood cells are eliminated. Then iron accumulates in the liver and later spreads to extrahepatic tissue such as endocrine tissues and the heart [49]. It has been demonstrated that iron overload can cause damage to bone marrow stem cells resulting in iron-correlated hematopoietic suppression, which is mediated by ROS-related signaling pathway [50, 51]. In turn, anemia caused by hematopoiesis inhibition makes further dependence on red-blood-cell transfusions, thus creating a vicious cycle.

### Alternations of iron metabolism in leukemia at cellular levels

TfR1, also known as CD71, is essential for iron uptake. Leukemia cells have increased expression of TfR1 compared to their normal counterparts and TfR1 is involved in the clonal development of leukemia [9, 52]. The expression of TfR1 is more prevalent in AML than that in ALL [53]. Moreover, poorly differentiated primary AML blasts tend to express higher levels of TfR1 than partially differentiated AML blasts [52]. TfR1 expression is higher in patients with T-cell ALL than patients with B-cell ALL [11, 54]. Clinical analysis also shows that overexpression of TfR1 in ALL is an adverse prognostic factor [11]. Transferrin receptor 2 (TfR2), another receptor for Tf, is also overexpressed in AML compared with normal counterparts [55]. Although both TfR1 and TfR2 are highly expressed in AML, only TfR2 levels were significantly associated with serum iron [56]. However, elevated mRNA levels of TfR2-α but not TfR1 or TfR2-β contribute to a better prognosis for AML patients [56]. It may be that TfR2-α increases the sensitivity of leukemia cells to chemotherapy drugs through an iron-independent pathway. The interaction of Tf with TfR can be modulated by HFE protein, thereby limiting the amount of internalized iron. Recent research suggests that *HFE* gene variants confer increased risk of leukemia that is attributed to the toxic effects of higher levels of iron [10, 57, 58]. In addition, the STEAP proteins function as ferric reductases that stimulate cellular uptake of iron through TfR1 [59]. Analysis of publicly available gene expression data shows that the STEAP1 is significantly overexpressed in AML which is associated with poor overall survival [60].

Transferrin-independent iron is also associated with iron overload in leukemia [61]. Lipocalin 2 (LCN2), also known as neutrophil gelatinase-associated lipocalin, is a less well studied protein that participates in iron uptake [62]. It is reported that overexpression of LCN2 was found in patients with AML, ALL, CML and CLL [63–67]. LCN2 is indispensable for BCR-ABL-induced leukomogenesis in the mouse model and involved in damaging normal hematopoietic cells [67]. Paradoxically, the analysis of whole-genome expression profiles from patients with leukemia (including AML, ALL and CLL) shows that LCN2 is downregulated at both mRNA and protein levels compared with healthy controls [64, 68]. The expression levels of LCN2 in the bone marrow of AML patients are lower than that of normal controls [69]. Importantly, the levels of LCN2 increased when AML patients achieved complete remission (CR), and decreased in patients with refractory disease [69]. Those data suggest that LCN2 expression is associated with better prognosis in AML. Therefore, further research is needed to clarify the specific function of LCN2 in different types of leukemia.

In addition to the abnormality of iron absorption, dysregulation of the iron-storage protein- ferritin also contributes to the pathogenesis and progression of leukemia. Ferritin is composed of two subunit types, termed ferritin heavy chain (FTH) and ferritin light chain (FTL) subunits. The c-MYC protein encoded by the proto-oncogene *c-MYC* is a transcription factor that activates the expression of iron regulatory protein-2 (IRP2) and represses ferritin expression [70]. IRP2 can bind to IREs, which results in increased synthesis of TfR1. The consequent increase in iron uptake and reduction in iron storage could raise the intracellular LIP level for metabolic and proliferative purposes^102^. It has been suggested that *c-MYC* gene plays an important role in the pathogenesis of lymphocytic leukemia [71]. T lymphocytic leukemia can be induced by the aberrant expression of *c-MYC* gene in the zebrafish model [72]. The suppression of *c-MYC* gene prevents leukemia initiation in mice, and reducing expression levels of *c-MYC* gene inhibits cell growth in refractory and relapsed T-cell acute lymphoblastic leukemia (T-ALL) [73]. FTH is also involved in the NF-κB signaling pathway-mediated cell proliferation, due to that FTH prevents ROS accumulation by iron sequestration, thereby inhibiting the pro-apoptotic c-Jun N-terminal kinase (JNK) signaling pathway [74]. It is reported that FTH and FTL are overexpressed in both AML cells and leukemia stem cells compared with normal HSCs regardless of genetic subgroups [40]. Thus, either downregulation or upregulation of ferritin contributes to the pathogenesis and progression of leukemia.

Studies have shown that cancer cells increase metabolically available iron not only by increasing iron uptake and regulating iron storage, but also by reducing iron efflux [7]. Accumulating evidence suggests that iron efflux mediated by FPN1 and controlled by hepcidin is involved in the development and progression of leukemia [43, 75, 76]. The expression level of FPN1 was decreased in the majority of AML cell lines, primary AML samples and leukemia progenitor and stem cells [76]. Low levels of FPN1 in AML are associated with good prognosis, which may occur due to the increased sensitivity to chemotherapy [75]. Of note, leukemia cells may synthesize hepcidin initiating a local autocrine signaling to degrade membrane FPN1, which needs to be confirmed by further research [77].

### Therapeutic opportunities of targeting iron metabolism in leukemia

**Fig. 2:**
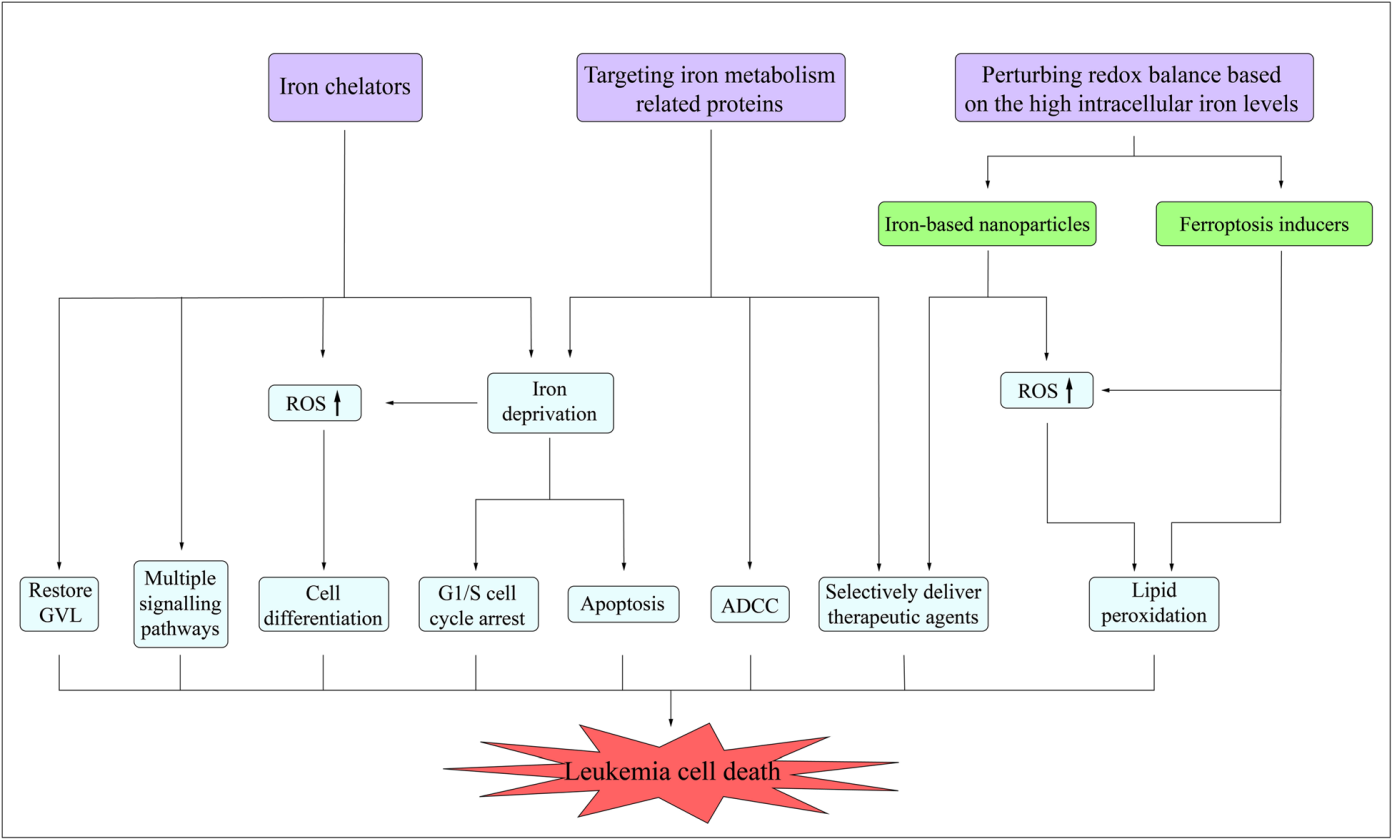
Therapeutic opportunities of targeting iron metabolism in leukemia cells. Iron deprivation by iron chelators or targeting iron metabolism related proteins induces differentiation, apoptosis and cell cycle arrest in leukemia cells. The generation of ROS is involved in the process of inducing cell differentiation. Iron chelators also play anti-leukemia roles through iron-independently regulating multiple signaling pathways or restoring GVL. ADCC is also involved in the anti-leukemia effect of targeting iron metabolism related proteins. Iron metabolism related proteins-targeted delivery systems or iron-based nanoparticles can selectively deliver therapeutic agents into leukemia cells to play enhanced anti-leukemia activity. Furthermore, iron-based nanoparticles elevate iron-catalyzed ROS levels, leading to increased cytotoxicity. Ferroptosis inducers perturb redox balance based on the high intracellular iron levels to induce ferroptosis in leukemia cells

As previously discussed, iron metabolism is dysregulated in patients with AL, which contributes to the development and progression of leukemia. These findings lead to the exploration of therapeutic approaches of targeting iron metabolism, including iron chelators, targeting iron metabolism related proteins and perturbing redox balance based on the high intracellular iron levels (Fig. 2).

### Iron chelators

Iron chelators are natural or synthetic small molecules that can decrease levels of intracellular iron by binding iron with a high affinity and promoting iron excretion. Several iron chelators, such as deferoxamine (DFO) and deferasirox (DFX), are clinically used to treat iron overload including secondary iron overload caused by repeated blood transfusions in patients with leukemia [78, 79]. Application of iron chelators has been proposed as an alternative anti-leukemia therapy in recent years [80]. Iron chelators exert anti-leukemia activity through several mechanisms, including lowering the LIP of leukemia cells by chelating intracellular iron, increasing ROS levels and activating MAPK and some other signaling pathways [14, 81, 82] (Table 1). The application of iron chelators in patients with leukemia and transfusional iron overload has dual effects of anti-leukemia and reducing the complications associated with iron overload.

**Table 1:** Summary on the role of iron chelators in leukemia

Name	Properties	Type of leukemia	Mode of action	Ref.
DFO	FDA-approved iron chelator	AML, ALL	Inhibits proliferation, induces apoptosis, differentiation and G1/S cell cycle arrest; inhibits ribonucleotide reductase, decreases the cyclin-dependent kinase inhibitor p21^CIP1/WAF1^ protein, induces ROS generation, activates IFN-γ/STAT1 and MAPK pathway.	[14, 80, 83–85]
DFX	FDA-approved iron chelator	AML, ALL	Inhibits proliferation and induces differentiation; induces ROS generation, inhibits NF-κB and mTOR signaling pathway, restores GVL.	[14, 16, 79, 86, 87]
3-AP	3-aminopyridine-2-carboxaldehyde thiosemicarbazone	AML, ALL	Inhibits ribonucleotide reductase.	[88]
SIHA	Tridentate iron chelator	AML	Induces apoptosis, cell cycle arrest and dissipation of the mitochondrial membrane potential.	[89]
Dp44mT	Di-pyridylketone thiosemicarbazone	AML, ALL	Induces apoptosis and G1/S cell cycle arrest; activates MAPK pathway.	[90]
EP	Thrombopoietin receptor agonist	AML	Induces differentiation and G1 cell cycle arrest.	[15]
CPX	Fungicide	AML, ALL, CML	Inhibits ribonucleotide reductase.	[91, 92]

Iron chelators effectively induce cell growth arrest and apoptosis in leukemia cells in a dose- and time-dependent manner [14, 16, 93]. Leukemia cells are more sensitive to iron chelators than their normal counterparts, most probably because their rapid proliferation depends on iron. Moreover, supplementation with iron attenuates the anti-leukemia effect of iron chelators, indicating that iron deprivation is one of the anti-leukemia mechanisms of iron chelators [16, 83]. It has been known for a long time that the rate-limiting step in DNA synthesis is catalyzed by ribonucleotide reductase whose catalytic activity is dependent on the continual presence of iron [94]. Iron deprivation blocks the synthesis of deoxyribonucleotides to inhibit proliferation in leukemia cells [84]. In consistent with the inhibition of DNA synthesis, iron deprivation appears to induce G1/S cell cycle arrest in leukemia cells [95]. Additionally, iron chelation decreases the cyclin-dependent kinase inhibitor p21^CIP1/WAF1^ protein through post-transcriptional regulation to achieve G1/S cell cycle arrest and induce apoptosis [96]. The mitogen-activated protein kinase (MAPK) pathway and the caspase pathway are also involved in the cell cycle arrest and apoptosis induced by iron depletion [16, 82].

Given the importance of iron in generation of free radicals and the critical role of ROS in HSCs metabolism, the role of ROS in anti-leukemia effects of iron deprivation has been studied [97]. Although iron deprivation by iron chelators may decrease ROS by reducing substrates for Fenton reaction, some iron chelators were shown to induce generation of ROS in a dose and time-dependent manner [85, 98]. Importantly, iron deprivation induces the differentiation of leukemia blasts and normal bone marrow precursors into monocytes/ macrophages by increasing ROS levels [14, 85, 95]. Iron deprivation–induced differentiation depends on activation of the downstream signaling pathways of oxidant stress response, including the MAPK/JNK signaling pathway [14, 86].

Iron chelators may play anti-leukemia roles through iron-independently regulating multiple signaling pathways related to cell survival. DFO induces apoptosis in T-ALL cells by reinstating the activation of interferon-γ (IFN-γ) /signal transducer and activator of transcription 1 (STAT1) pathway which is attenuated in T-ALL cells shielding them from the anti-proliferative effect of IFN-γ [99]. DFX also exerts its anti-leukemia activity by inhibiting extracellular signal-regulated kinase (ERK) phosphorylation, repressing the mammalian target of rapamycin (mTOR) and NF-κB signaling pathway [81, 100, 101].

Iron chelators not only have anti-leukemia effects singly, but also exhibit synergistic anti-leukemia effects when combined with traditional chemotherapy drugs. DFO increases the sensitivity of human myeloid leukemia cells to doxorubicin (DOX) and arabinoside cytosine (Ara-C) [102, 103]. DFO combined with arsenic trioxide (ATO) has synergistic effects on anti-proliferation and inducing apoptosis in APL [104]. DFO can be synergized with L-asparaginase or dexamethasone to decrease survival of leukemia cells or associated with DNA-damage inducing agents to increase apoptosis in T-ALL [9]. DFX shows synergistic effect with the DNA methyl transferase inhibitor decitabine (DAC) on apoptosis and cell cycle arrest in leukemia cell lines [88]. However, it has been suggested that DFX creates a synergistic effect combined with Ara-C, while antagonizes the anti-leukemia effect of DOX in the treatment of AML [89]. Therefore, further studies are needed to confirm the effects of iron chelators combined with different traditional chemotherapy drugs to provide information on how to select drug combination for the treatment of leukemia in future clinical trials.

In addition to traditional iron chelating agents, some new iron chelators have been developed to improve the bioavailability and have also been identified to play anti-leukemia roles. For example, Triapine (3-AP) decreases the DNA synthetic capacity of circulating leukemia cells when administered in patients with refractory leukemia [105]. Salicylaldehyde isonicotinoyl hydrazine analogues (SIHA) is reported to dose-dependently induce apoptosis, cell cycle arrest and dissipation of the mitochondrial membrane potential in AML cells [90]. Additionally, the synthetic chelator di-2-pyridylketone-4,4,-dimethyl-3-thiosemicarbazone (Dp44mT) shows a significantly high affinity with Fe^2+^ and allows bound iron to participate in redox reactions and free radical formation [91]. Dp44mT has been demonstrated to inhibit the proliferation of leukemia cells with a G1/S phase arrest, accompanied by caspase-mediated induction of apoptosis [106]. Importantly, several agents used in clinical practice for other indications have also been discovered to function as iron chelators. Eltrombopag (EP), a small-molecule nonpeptide thrombopoietin receptor agonist, is reported to block the cell cycle in G1 phase and induce differentiation of leukemia cells through reducing free intracellular iron [15]. The antimicrobial ciclopirox olamine (CPX) has been identified to functionally chelate intracellular iron, which is important for its anti-leukemia cytotoxicity [107]. Further study demonstrates that iron chelation of CPX mediates inhibition of Wnt/β-catenin signaling and thus reduces expression of the Wnt target gene AXIN2 in leukemia cells of patients with AML [87].

Iron chelators have also shown promising anti-leukemia effects in human trials. A 73-year old male patient with relapsed, refractory acute monocytic leukemia achieved hematological and cytogenetic CR after application of DFX with no additional chemotherapy for 12 months [108]. Moreover, a 69-year-old male patient with relapsed AML had decreased peripheral blast counts accompanied by increased monocytic differentiation and partially reversed pancytopenia after DFO and vitamin D therapy [14]. In addition to AML, a six weeks old infant with ALL, who failed to attain remission with induction chemotherapy (IC), had peripheral blast counts significantly reduced accompanied by myelomonocytic differentiation after treatment with DFO and Ara-C [93]. In addition to these sporadic success stories, some clinical trials have also demonstrated the anti-leukemia effect of iron chelators (Table 2, refer to the website: https://clinicaltrials.gov/). A retrospective case control study has shown that DFO administration after allo-HSCT in patients with hematological malignancies reduced relapse incidence and improved disease-free survival [109]. A pilot clinical trial showed that DFO administration prior to allo-HSCT in patients with AL or MDS resulted in good outcomes, with no death or relapse, at a median follow-up of 20 months [110]. Similarly, a retrospective observational study of 339 patients demonstrates that the oral chelator DFX significantly reduces relapse mortality and restores graft-vs-leukemia effects (GVL) after allo-HSCT in AML, which is evidenced by high proportion of NK cells and suppressed regulatory T cells in peripheral blood [111]. Importantly, studies have shown that DFX, at concentrations equal to those clinically used or even at higher ones, has no harm to the viability of normal HSCs [85, 112]. DFX is even reported to have a beneficial effect on the hematopoietic recovery in patients after allo-HSCT [113]. A multicenter prospective cohort study (PCS) on the impact of DFX on relapse after allo-HSCT in patients with AML is recruiting (NCT03659084). Moreover, a randomized controlled trial (RCT) and a single group assignment (SGA) clinical trial have also been registered to clarify the effect of DFX on response rate of AL patients who are not fit for standard chemotherapy regimens (NCT02413021, NCT02341495). Those clinical trials will more strongly demonstrate the effect of DFX on the treatment of leukemia and post-transplant hematopoiesis.

**Table 2:** Basic characteristics of clinical trials on iron chelators in the treatment of leukemia

Name	Trial ID	Status	Design	N	Condition	Treatment	Outcome (/Measures)
DFO	NCT00658411	Terminated	SGA	5	AL, MDS	DFO (50 mg/kg/d) for ≥2 weeks prior to HSCT.	At a median follow-up of 20 months, no patient relapsed or died. Estimated 2-year OS and PFS are both 100%. No patient developed grade III/IV acute GVHD or VOD.
DFX	NCT03659084	Recruiting	PCS	150	AML, MDS	DFX (10 mg/kg/d) at 6 months after allograft, for 3–6 months.	RFS (at 2 years), cumulative incidence of GVHD (at 3 months, 1 and 2 years) and toxicity of DFX (an average of 4 years).
	NCT02413021	Unknown	RCT	40	AL	Ara-C (20 mg/m^2^ bid, for 10 days, repeated every 30 days) with or without DFX (20 mg/kg/d)	CR or PR (at first month).
	NCT02341495	Unknown	SGA	29	AML (age ≥ 65 years)	DFX (20 mg/kg/d) with VD3 (4000 IU/d) and Azacitidine (75 mg/m^2^/d) on d1–7, repeated every 28 days for 8 cycles.	CR, OS, PFS and DOR (up to 5 years).
3-AP	NCT00064090	Completed	Ph-I	32	AL, MDS	3-AP (105 mg/m^2^/d) followed by Ara-C (100–800 mg/m^2^/d) on days 1–5, repeated every 21 days for up to 6 courses in the absence of PD or toxicity.	Of 31 evaluable patients, 4 (13%) achieved a CR. The median DOR for responders was 36 weeks. The median OS for all patients and responders was 30.9 weeks and 12.6 weeks, respectively. DLTs included mucositis, neutropenic colitis, neuropathy and hyperbilirubinemia.
	NCT00077181	Completed	Ph-I	25	AML, CML-AP	Ara-C (100 mg/m^2^/d, d1–5) and 3-AP (50/75/100 mg/m^2^/d, d2–5), repeated every 28 days for up to 4 courses in the absence of PD or toxicity.	The OR rate was 3/25, with a CR rate of 2/25. An elderly patient with primary refractory AML had HI. DLTs included methemoglobinemia, cerebellar toxicity, sensorimotor peripheral neuropathy and mucositis.
	NCT00077558	Completed	Ph-I	33	AL, MPD	Group A: 3-AP (105 mg/m^2^/d, d1–5) followed by fludarabine (15–30 mg/m^2^/d, d1–5); Group B: 3-AP (200 mg/m^2^, d1) followed by fludarabine (15–30 mg/m^2^/d, d1–5); repeated every 21 days until PD or toxicity.	CR and PR occurred in group A (5/24, 21%), with CR occurring at the 2 highest fludarabine doses (2/12, 17%). No CR or PR occurred in group B. Response durations were short and ranged from 1.5 to 7 months. DLTs included fever, methemoglobinemia and metabolic acidosis.
	NCT00381550	Completed	SGA	37	sAML, CML-BP, MPD	3-AP (105 mg/m^2^/d) followed by fludarabine (30 mg/m^2^/d) on d1–5, repeated every 21 days until PD or toxicity.	The OR rate was 49% (18/37), with a CR rate of 24% (9/37). In sAML, the OR rate and CR rate were 48 and 33%, respectively. Median OS of the entire cohort was 6.9 months, with a median OS of overall responders of 10.6 months.
CPX	NCT00990587	Completed	Ph-I	23	AL, CML, CLL, MDS, Hodgkin’s Disease	CPX (5–80 mg/m^2^/d d1–5, once daily), repeated every 21 days, or CPX (80 mg/m^2^/d d1–5, four times daily); repeated every 21 days for multiple cycles in the absence of PD or toxicity.	No patients achieved a CR or PR, but HI was observed in 2 patients. Disease stabilization occurred in 5 additional AML patients and 1 MDS patient. DLTs were gastrointestinal toxicities and knee pain.
EP	NCT00903422	Completed	RCT	98	MDS, sAML/MDS	EP (50-300 mg/d) or placebo until PD or toxicity.	No patients had a CR, but two (3%) patients in the EP group had PR. Median OS and PFS were longer in the EP group than in the placebo group (27.0 weeks vs 15.7 weeks, 8.1 weeks vs 6.6 weeks, respectively). HI was recorded in 23 (36%) EP patients and eight (24%) placebo patients. PD was recorded in 40 (63%) patients in the EP group and 22 (65%) patients in the placebo group. The incidence of drug-related adverse events of grade 3 or higher were similarly in the two groups.
	NCT01890746	Completed	RCT	149	AML (except M3 or M7)	IC: daunorubicin (90 mg/m^2^/d, 60 mg/m^2^/d for age > 60 years, d1–3) and Ara-C (100 mg/m^2^/d, d1–7); with EP (200 mg/d, 100 mg/d for east Asians) or placebo until PLT ≥200 × 10^9^/L, or remission, or after 42 days from the start of IC.	The EP group and the placebo group achieved a similar OR rate (70% vs 73%), and so did the CR rate and PR rate. Median DOR was longer in the placebo group than in the EP group (not reached vs 22 months). Median OS was shorter in the EP group than in the placebo group (15.4 months vs 25.7 months), and more patients died in the EP group. The incidence of LVEF events and the frequency of AE were similar in both groups during IC. However, there was a trend for more serious AE, including fatal AE, in the EP group.
	NCT03603795	Recruiting	RCT	110	AML (age > 60 years, except M3 or M7)	IC (daunorubicin 60 mg/m^2^/d d1–3; Ara-C 100 mg/m^2^/d d1–7 and Lomustine 200 mg/m^2^ d1), with EP (200 mg/d, 100 mg/d for east Asians) or placebo from d11 to response evaluation or PLT > 100 × 10^9^/L (maximum to d45).	OR rate and percentage of patients with PLT > 100 × 10^9^/L (at d45), OS and RFS (at 1 year), OS (at 2, 3 and 5 years).
	NCT02446145	Unknown	RC	238	AML (age ≥ 65 years, except M3)	Decitabine (20 mg/m^2^/d d1–5, repeated every 28 days) with EP or placebo (200 mg/d from d1, 100 mg/d for east Asians, and dose modification up to 300 mg/d, 50–150 mg for east Asians).	OR, OS, RFS and treatment change-free survival (up to 4 years).

Refer to the website: https://clinicaltrials.gov/

There are also some clinical trials to study the safety and the anti-leukemia effect of new iron chelators. A dose-escalating phase I study (Ph-I) showed that 4 out of 31 patients (the majority with refractory AL) achieved a CR with a longer median survival after treatment with 3-AP and Ara-C [114]. Dose-limiting toxicities (DLTs) in the study were mucositis, neutropenic colitis, neuropathy and hyperbilirubinemia [114]. In another Ph-I study, similar DLTs were also observed and the toxicities of combination of 3-AP and Ara-C were similar to that of Ara-C singly at the same dose and schedule [115]. 3-AP followed by the adenosine analog fludarabine in adult patients with refractory AL showed controllable drug-related toxicities, including fever, methemoglobinemia and metabolic acidosis [116]. In a single group assignment (SGA) phase II trial in patients with secondary AML (sAML), chronic myeloid leukemia in blast phase (CML-BP) or MPD, 3-AP followed by fludarabine achieved an overall response (OR) rate of 49% (18/37), with a CR rate of 24% (9/37), which further demonstrates the promise of 3-AP to be clinically applied in the treatment of leukemia [117]. A phase I study of CPX showed that once-daily dosing was well tolerated in patients with relapsed or refractory AML and 2 patients had hematologic improvement (HI) while no patients achieved complete remission or partial remission (PR) [107]. The thrombopoietin receptor agonist EP has been approved for the treatment of patients with chronic immune thrombocytopenia and refractory severe aplastic anemia. The role of EP in patients with leukemia has been investigated in several clinical trials. A multicenter RCT reported that EP had an acceptable safety profile in patients with advanced MDS or sAML/MDS (secondary acute myeloid leukemia after myelodysplastic syndrome) and 2 (3%) patients achieved PR [118]. However, data from another multicenter RCT do not support combining EP with IC in patients with AML [119]. The addition of EP didn’t improve the disease response, but there was a shorter OS and a trend for more serious adverse events (AE) in the EP group [119]. Further clinical studies, conducted in larger patient populations with more rigorous design are ongoing to assess the safety and the use of EP in elderly patients with AML, except M3 or acute megakaryocytic leukemia (M7) (NCT03603795; NCT02446145).

Current preclinical and clinical studies have confirmed the anti-leukemia effect of both traditional iron chelating agents and some new iron chelators. Notwithstanding the wide use of traditional iron chelating agents in the treatment of iron overload caused by repeated blood transfusions, the optimal doses for anti-leukemia treatment and their safety remain to be further studied. Systematic studies, which evaluate not only the toxicity but also the anti-leukemia effect of those new iron chelators in different subtypes of leukemia are also needed. More research will focus on the combination effect of iron chelators with different chemotherapeutic agents and the best scheme of their combination to bring to fruition their application in the clinical management of leukemia.

### Targeting iron metabolism related proteins

In addition to iron chelators, depletion of intracellular iron can be achieved by targeting iron metabolism-related proteins. As a receptor that is critical for cellular iron uptake, TfR is an attractive target for depleting intracellular iron of leukemia cells. Both inhibitory and non-inhibitory anti-TfR monoclonal antibodies result in decreased Tf binding sites and subsequently inhibit Tf uptake, leading to growth inhibition in leukemia cells by iron deprivation [120]. A24, a monoclonal antibody directed against TfR1, competitively inhibits Tf binding to TfR1 and induces TfR1 endocytosis in lysosomal compartments where the receptor is degraded [121]. A24 inhibits proliferation and induces differentiation of leukemia cells by depleting the intracellular iron [14, 121, 122]. Combinations of two or more anti-TfR monoclonal antibodies can interact synergistically to play anti-leukemia effects, which correlates with their ability to block Tf-mediated iron uptake [123]. When combined with DFO, the monoclonal antibodies against TfR produce greater damage to iron uptake and a rapid depletion of iron pools [83, 124]. In addition to the deprivation of intracellular iron, JST-TfR09, an IgG monoclonal antibody to human TfR1, also plays an anti-leukemia effect through antibody-dependent cell-mediated cytotoxicity (ADCC) [125]. Though anti-TfR monoclonal antibodies show promising effects in the treatment of leukemia in those preclinical studies, there are some limitations for their clinical application. TfR is not specifically expressed in leukemia cells, it is also displayed by a wide variety of normal tissues. Depression of stem cell activity in bone marrow and altered distribution of red blood cell progenitors were observed in leukemia-bearing mice after receiving repeated injections of anti-TfR antibody [126]. A phase I trial of IgA monoclonal anti-TfR antibody 42/6 showed that 42/6 was generally well tolerated, although only transient, mixed antitumor responses were observed in patients with hematological malignancies [92]. Nevertheless, 42/6 also induced apparent down-regulation of TfR display by bone marrow cells, which could impair production of red blood cells [92]. These observations raised major concerns for the use of anti-TfR antibodies that maturing erythroid cells would be severely affected by anti-TfR antibodies, leading to anemia.

Taking the upregulation of the TfR on the leukemia cell surface into account, various TfR-targeted delivery systems consisting targeting ligands, carriers, and therapeutic agents have been developed. Not only to mention that TfR expression is significantly upregulated on leukemia cells, the binding of ligands to TfR also elicits very effective receptor-mediated endocytosis [127]. The ligands targeting TfR mainly include Tf, monoclonal antibodies, single-chain antibody fragment (scFv) and targeting peptides. Initially, these ligands are directly linked to some therapeutic agents. Conjugating artemisinin to a TfR targeting peptide shows anti-leukemia activity with a significantly improved leukemia cell selectivity [128]. With the development of technology, some carriers have been developed to link ligands and therapeutic agents for improving the efficacy and safety in therapeutic agent delivery, among which liposomes, dendritic molecules and nanoparticles have been widely used [129, 130]. A human serum albumin based nanomedicine, which is loaded with sorafenib and conjugated ligands for TfR specific delivery, can play enhanced anti-leukemia activity in drug resistant CML patient samples [130]. The sensitivity of leukemia cells to imatinib can also be enhanced by encapsulated with TfR targeted liposomes [131]. It has been reported that anti-TfR-coupled liposomes are more effective for intracellular drug delivery to T-ALL cells than anti-Tac conjugates, a monoclonal antibody directing against the interleukin-2 receptor [129]. Tf conjugated lipopolyplexes carrying G3139, an antisense oligonucleotide for B-cell lymphoma-2 (Bcl-2), induce remarkable pharmacological effect of Bcl-2 inhibition in AML cells and are more effective than free G3139 or non-targeted lipid nanoparticles [132]. Furthermore, iron chelator DFO can up-regulate TfR expression in leukemia cells, resulting in a further increase in anti-leukemia effect of TfR-targeted lipid nanoparticles carrying G3139 [133]. Because traditional chemotherapy drugs are difficult to pass the blood-brain barrier, leukemia cells sheltered in the central nervous system become the source of extramedullary recurrence of leukemia. Accumulating evidences have suggested that TfR-targeted delivery systems are promising strategies in enhancing the blood-brain barrier penetration [134]. More clinical trials of TfR-targeted delivery systems are expected to further improve their therapeutic potential.

In addition to TfR, other iron metabolism related proteins are also promising therapeutic targets. It has been suggested that STEAP can be targeted by specific CD4^+^T cells in non-small-cell lung carcinoma [135]. This provides a basis for STEAP to be used as an immunotherapy target for leukemia. Targeting ferritin results in dramatic anti-leukemia effect, suggesting that the pharmacological modulation of the storage protein of iron could be a new therapeutic target in leukemia [136]. Another consideration is that secreted ferritin can be absorbed by the TfR. Ferritin has also been commonly used for drug targeting because of its nanocage structure, which make it possible to deliver anti-leukemia drugs in the future [137]. Such naturally occurring structure is superior to synthetic ones due to its low toxicity and negligible immune responses. It’s reported that c-MYC contributes to drug resistance in AML and inhibition of c-MYC induces differentiation, apoptosis, and cell cycle arrest in leukemia cells [138, 139].

It appears logic to apply approaches targeting iron-associated proteins as therapeutic measures due to their expression differences between normal cells and leukemia cells. However, monoclonal antibodies targeting iron-associated proteins may also damage normal cells, especially those with high iron demand, because iron-associated proteins are not specific in leukemia cells. To conquer the limitations associated with conventional chemotherapy, TfR or ferritin targeted drug delivery systems have been introduced. Furthermore, the combination of those drug delivery systems and molecular targeted drugs brings hope to increase drug efficacy and alleviate the toxicity caused by non-specificity of iron metabolism-related proteins. As prospective clinical data is still missing, approaches to targeting iron-associated proteins are still far from being usable for leukemia treatment.

### Perturbing redox balance based on the high intracellular iron levels

#### Ferroptosis and Ferritinophagy

Ferroptosis is a form of oxidative cell death, which is characterized by the production of ROS from accumulated iron and lipid peroxidation to trigger death [1, 140]. As iron is crucially involved in the formation of ROS, iron-catalyzed ROS production is primarily responsible for ferroptosis [1, 141]. Iron chelator DFO and heat shock protein β-1 prevent ferroptosis through reducing intracellular iron, but increasing intracellular iron promotes ferroptosis [140, 142, 143]. Ferritinophagy is an autophagic phenomenon that selectively degrades ferritin to release intracellular free iron and thus promotes ferroptosis [144]. Due to the importance of ROS in ferroptosis, antioxidants are critical regulators of ferroptosis. Glutathione peroxidase 4 (GPX4), which is the only antioxidant enzyme known to directly reduce lipid peroxides produced by ROS, plays a pivotal role in ferroptosis [145, 146]. It has been identified that regulation of GPX4 is a common mechanism shared by multiple ferroptosis inducers [145]. One class of ferroptosis inducers such as RSL3 inhibits GPX4 directly [145]. As glutathione (GSH) is a cofactor essential for GPX4 function, inhibition of GPX4 function by depleting GSH can also induce ferroptosis [146]. Because GSH production is limited by the availability of cystine/cysteine, another class of ferroptosis inducers (such as erastin, sorafenib) reduces GSH production through inhibiting cystine uptake by system X_c_^−^, a cell surface cysteine-glutamate antiporter [140, 145, 147]. The well-known tumor suppressor p53 acts as a positive regulator of ferroptosis by inhibiting the expression of SLC7A11, a key component of system X_c_^−^[148]. The mechanism of ferroptosis triggered by the multikinase inhibitor sorafenib includes not only inhibition of system X_c_^−^, but also iron-dependent induction of oxidative stress [147, 149].

**Fig. 3:**
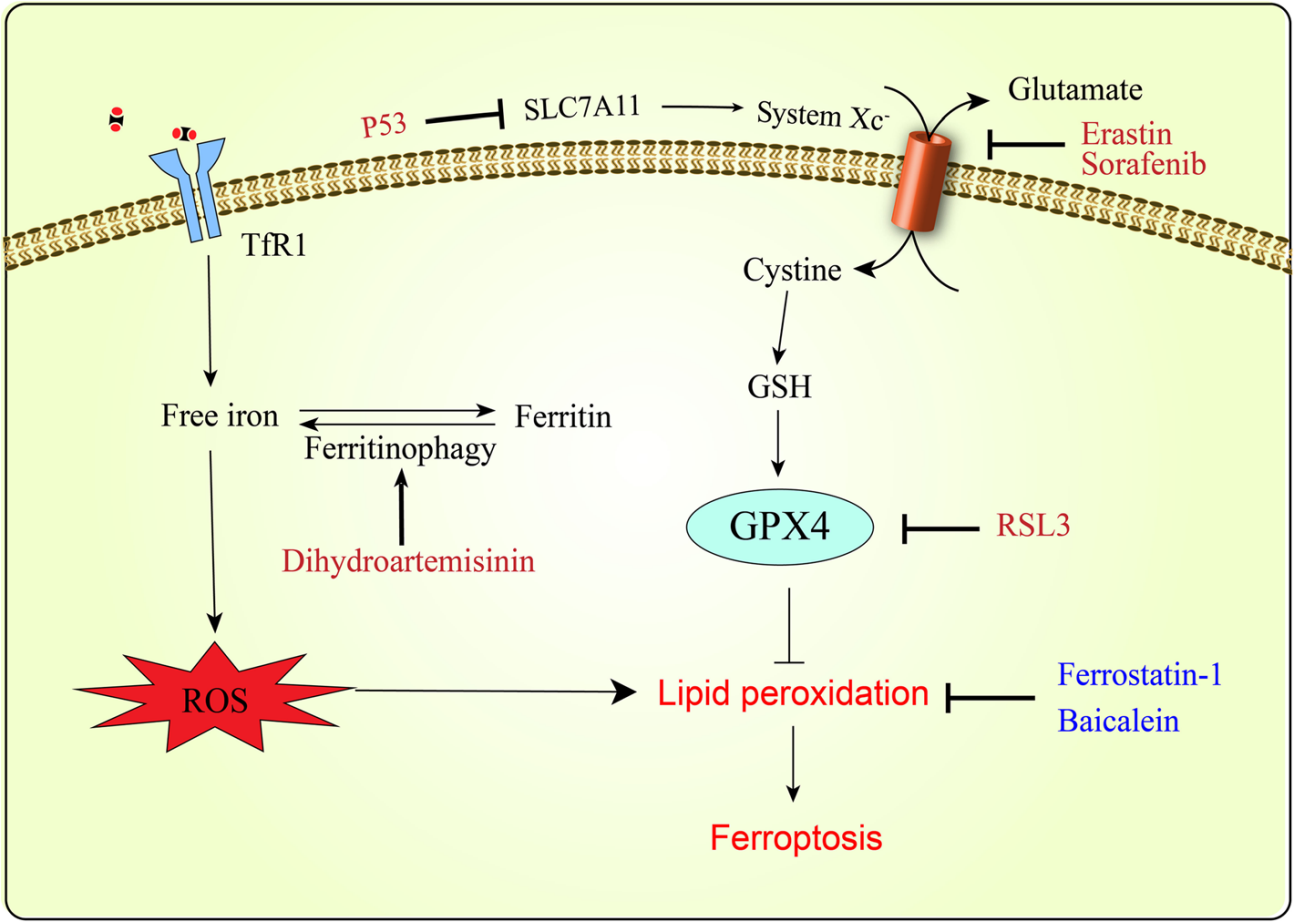
Schematic model of ferroptosis in leukemia cells. Ferroptosis occurs as a result of iron-mediated oxidative stress and lipid peroxidation-mediated cytotoxicity. It could be due to elevated intracellular iron concentration or inhibition of GPX4 activity. Dihydroartemisinin induce ferroptosis by ferritinophagy and subsequent accumulation of ROS. RSL3 inhibits GPX4 directly, while erastin, sorafenib and p53 decrease GSH production by inhibiting cysteine transport. Lipoxygenase inhibitors (such as Ferrostatin-1 and Baicalein) suppress ferroptosis through inhibiting lipid peroxidation

Recently, triggering ferroptosis based on the high intracellular iron levels has become a promising therapy to preferentially target leukemia cells (Fig. 3). The tumor suppressing function of ferroptosis has been identified in a wide range of malignancies, including fibrosarcoma, prostate carcinoma, osteosarcoma and so on [140, 145, 150]. Recent studies have indicated that RSL3 or Erastin can trigger death in leukemia cells and even enhance the sensitivity of leukemia cells to chemotherapeutic agents [151–153]. In turn, lipoxygenase inhibitors (such as Ferrostatin-1 and Baicalein) can protect ALL cells from ferroptosis [153]. The ferroptosis inducer sorafenib has been clinically approved for the treatment of FLT3-ITD mutated AML, whose mechanism may include induction of ferroptosis in AML cells [154, 155]. Artemisinin and its derivatives are widely used to treat multidrug-resistant malaria due that they owe the endoperoxide bridge and can induce ROS production in the presence of iron [156]. It has been recently suggested that dihydroartemisinin can induce ferroptosis in leukemia cells through ferritinophagy which increases the cellular LIP and thus promotes accumulation of ROS [157, 158]. The naturally occurring compound ardisiacrispin B and epunctanone have also been identified to induce ferroptosis in ALL cells [159, 160]. Therapies by inducing ferroptosis and ferritinophagy possess great potential in leukemia treatment. In the future, more and more research will focus on disturbing the redox balance to increase sensitivity of leukemia cells to chemotherapeutic agents.

#### Iron-based nanoparticles

More and more attention has been paid to the research of iron-based nanoparticle antitumor therapy [161]. The iron oxide nanoparticles are reported to induce apoptosis and cell cycle arrest at sub-G1 phase in T-ALL cells [162]. Furthermore, iron-based nanoparticles can release iron in the form of Fe^2+^ or Fe^3+^ which participates in the Fenton reaction and induce ferroptosis [163]. Ferumoxytol (feraheme), an intravenous preparation of iron oxide nanoparticles, is available for the treatment of iron deficiency in clinic [164]. It is recently reported that ferumoxytol shows an anti-leukemia effect due to increased iron-catalyzed ROS and low expression of the iron exporter FPN1 results in enhanced susceptibility of AML cells to ferumoxytol [76]. Besides, traditional chemotherapy drugs can be delivered by the iron-based nanoparticles for enhancing their anticancer efficacy. It is reported that the anti-leukemia effect of cytarabine is enhanced by being coated on Fe_3_O_4_@SiO2 nanoparticles [165].

The iron-based nanoparticles can be functionalized with active and passive targeting ability to reduce the adverse effects of iron-catalyzed ROS to normal cells. Satake N et al. composed nanocomplexes with super paramagnetic iron oxide nanoparticles, antiCD22 antibody and MAX dimerization protein 3 small interfering RNA molecules which showed cytotoxic effects to precursor B-cell ALL selectively and enhanced the anti-leukemia effect of chemotherapy drug vincristine or DOX [166]. The iron-based nanoparticles can also be manipulated by the magnetic field to accumulate preferentially at tumor sites as a result of the enhanced permeability and retention phenomenon [163]. It has also been suggested that the magnetic field has potential to increase the blood-brain barrier permeability of iron-based nanoparticles for therapy of various brain diseases [167]. Furthermore, the magnetic field itself can play anti-leukemia effects by increasing ROS production [168]. Therefore, the application of iron-based nanoparticles directed by magnetic field may provide an approach to the prevention and treatment of central nervous system infiltration of leukemia.

Even though iron-based nanoparticle systems with multiple function bring us a step closer to delivering personalized medicine into leukemia cells, there are still multiple obstacles to the clinical application of these new iron-based nanoparticle systems. Currently, the toxicity of iron-based nanoparticle systems is of great concerns. No observable toxicity is seen at low levels of iron-based nanoparticles, while the particles may trigger cellular stress, weaken inflammatory reactions, increase the expression of genes involved in cell signaling and thus impact signaling pathways in the case of high dose exposure [169]. It is critical to design functionalized iron-based nanoparticles which are able to meet the demands of a particular application and have good security in the human body. To inform the design of safe iron-based nanoparticles, a better understanding of the relationship between their toxicity with different surface properties, size, hydrophobicity, and release of iron ions is needed. It is expected that in the near future, iron-based nanoparticle systems, conjugated with new targeted drugs, could replace our current treatments and leukemia could become a nonfatal disease with good prognosis.

### Conclusions and prospects

Accumulating evidence implicates changes in iron metabolism as crucial features of leukemia. The alteration of iron metabolism in leukemia cells is generally associated with high iron requirements and high oxidative stress, suggesting that leukemia cells may be more vulnerable to changes in iron and ROS levels compared with normal cells. In addition to iron chelators and therapies targeting iron metabolism-related proteins, perturbing redox balance based on the high intracellular iron levels also has promising therapeutic implications for the treatment of leukemia. The application of ferroptosis and ferritinophagy in the treatment of leukemia is just beginning as a new way of death involving iron. With the development of nanotechnology, efforts to harness insights for therapeutic advantages of iron-based nanoparticles have begun. The magnetic fields not only concentrate nanoparticles, but also promote the production of ROS in cells to play anti-leukemia effects.

Though researches in the past few years have expanded our insights into the regulation of iron in leukemia and treatment strategies that target iron metabolism, more studies are warranted to fully clarify the specific mechanism that link iron, oxidative stress, and leukemia development. Efforts are still needed to optimize therapies for leukemia targeting towards iron metabolism. A recent study finds that iron depletion may influence the expression of Major Histocompatibility Complex class I molecules to increase the target susceptibility of cancer cells to NK cell recognition [170]. This provides a basis to kill leukemia cells through modulating immune system by iron depletion. Ascorbate is an essential nutrient commonly regarded as an antioxidant. However, high-dose ascorbate is demonstrated to induce hydrogen-peroxide-dependent cytotoxicity toward a variety of cancer cells without adversely affecting normal cells [171]. Hydrogen-peroxide generated by high-dose ascorbate reacts with excess intracellular iron to produce cytotoxic ROS in cancer cells. Ascorbate also suppress leukemogenesis by promoting Tet function in HSCs [172]. Therefore, ascorbate is a prospective anti-leukemia agent due to both its ability of perturbing redox balance based on the high intracellular iron levels in leukemia cells and activation of Tet enzymes. More and more attention will be attached to iron-based nanoparticles due to their multiple advantages. In the future, there will be strategic opportunities to enhance therapeutic efficacy by associating the iron-based nanoparticles with other components, such as ferroptosis inducers, some genes modulating the expression of iron metabolism related proteins, targeting small molecules and so on. It is appealing to combine efforts from different disciplines to pursue rational design of effective leukemia therapy strategies based on iron metabolism.

## Data Availability

Not applicable.

## Abbreviations

3-AP: Triapine
ADCC: Antibody-dependent cell-mediated cytotoxicity
AE: Adverse events
AL: Acute leukemia
ALL: Acute lymphoblastic leukemia
allo-HSCT: Allogeneic hematopoietic stem cell transplantation
AML: Acute myeloid leukemia
apo-Tf: Apo-transferrin
Ara-C: Arabinoside cytosine
ATO: Arsenic trioxide
Bcl-2: B-cell lymphoma-2
CLL: Chronic lymphoblastic leukemia
CML: Chronic myeloid leukemia
CML-AP: Chronic myeloid leukemia in the accelerated phase
CML-BP: Chronic myeloid leukemia in blast phase
Cp: Ceruloplasmin
CPX: Ciclopirox olamine
CR: Complete remission
DAC: Decitabine
Dcytb: Duodenal cytochrome b reductase
DFO: Deferoxamine
DFX: Deferasirox
DLT: Dose-limiting toxicity
DMT1: Divalent metal transporter 1
DOR: Duration of remission
DOX: Doxorubicin
Dp44mT: Di-2-pyridylketone-4,4,-dimethyl-3-thiosemicarbazone
EP: Eltrombopag
ERK: Extracellular signal-regulated kinase
FDA: Food and Drug Administration
Fe^2+^: ferrous iron
Fe^3+^: ferric iron
FPN1: Ferroportin 1
FTH: Ferritin heavy chain
FTL: Ferritin light chain
GPX4: Glutathione peroxidase 4
GSH: Glutathione
GVL: Graft-vs-leukemia
HEPH: Hephaestin
HFE: Hemochromatosis
HI: Hematologic improvement
HO-1: Heme oxygenase 1
HSC: Hematopoietic stem cell
IC: Induction chemotherapy
ID: Identifier
IFN-γ: Interferon-γ
IREs: Iron responsive elements
IRP2: Iron regulatory protein-2
IRPs: Iron regulatory proteins
JNK: C-Jun N-terminal kinase
LCN2: Lipocalin 2
LIP: Labile iron pool
LOX: Lipoxygenase
LVEF: Left ventricular ejection fraction
M3: Acute promyelocytic leukemia
M7: Acute megakaryocytic leukemia
MAPK: Mitogen-activated protein kinase
MDS: Myelodysplastic syndrome
MPD: Myeloproliferative disorders
MTD: Maximally tolerated dose
mTOR: Mammalian target of rapamycin
N: Number
NF-κB: Nuclear factor kappa-B
NTBI: Non-transferrin bound iron
OR: Overall response
OS: Overall survival
PBC: Peripheral blood cell
PCS: Prospective cohort study
PD: Progression disease
PFS: Progression-free survival
Ph-I: Dose-escalating phase I study
PLT: Platelet
PR: Partial remission
RCT: Randomized controlled trials
RFS: Relapse free survival
ROS: Reactive oxygen species
sAML: Secondary AML
sAML/MDS: Secondary acute myeloid leukemia after myelodysplastic syndrome
SGA: Single group assignment
SIHA: Salicylaldehyde isonicotinoyl hydrazine analogues
STAT1: Signal transducer and activator of transcription 1
STEAP: Six-transmembrane epithelial antigen of the prostate
T-ALL: T-cell acute lymphoblastic leukemia
Tf: Transferrin
TfR1: Transferrin receptor 1
TfR2: Transferrin receptor 2
VD3: Vitamin D3
VOD: Venoocclusive liver disease
